# How we learn to like: the role of perceptual learning in development of liking, quality perception, and appreciation

**DOI:** 10.1007/s10068-025-01857-4

**Published:** 2025-04-03

**Authors:** Jae-Hee Hong

**Affiliations:** 1 Department of Food and Nutrition, Seoul National University, Seoul, 08826 Republic of Korea; 2 Human Ecology Research Institute, Seoul National University, Seoul, 08826 Republic of Korea

**Keywords:** Perceptual learning, Liking, Repeated exposure, Appreciation, Quality

## Abstract

This review explores how perceptual learning contributes to the development of liking for novel and unfamiliar foods. Food acceptance evolves dynamically with repeated exposure. Repeated exposure significantly enhances the acceptance of novel and unfamiliar foods through perceptual changes, as evidenced by empirical studies and theoretical frameworks. Repeated exposure facilitates perceptual learning by enhancing the ability to detect specific sensory attributes and to filter out irrelevant features. The impact of perceptual learning is noticeable in the wine industry, where trained experts demonstrate superior detection, differentiation, and identification skills. This acquired perception influenced consumer acceptance, appreciation, and quality evaluation by providing richer attributes for constructing mental representations of food. However, investigating the mechanisms behind the effect of perceptual learning on liking is challenging, due to complex interplay between cognitive and perceptual inputs. Future studies should extend beyond wine to better understand how perceptual learning shapes food choice driven by liking and appreciation.

## Introduction

Liking for food is shaped not only by its sensory characteristics (such as taste, aroma, texture, appearance, and temperature) but also by how these sensory inputs are interpreted and processed through memory, experience, and knowledge (Cardello, 1994; Piqueras-Fiszman and Spence, 2015). Objects and stimuli are perceived within a specific frame or context (Chung, 2019). A frame of reference, shaped by past experiences, plays a significant role in how sensory characteristics are perceived and how food preferences are formed (Ishii and O’Mahony, 1991; Lawless and Heymann, 2010).

Particularly, pre-existing knowledge about food shapes strong expectations about the sensory experiences during consumption. These expectations are typically based on past experiences and memories related to the food, the sensory information perceived while eating it, and inferences drawn from similar or related experiences (Oliver and Winer, 1987). Sensory perception and preference may shift based on whether the actual experience meets, exceeds, or falls short of these expectations. These changes are explained by the assimilation/contrast model (Cardello, 2007; Deliza and MacFie, 1996). A small gap between expectation and actual experience results in assimilation, where the perceived characteristics or preferences adjust to closely match the expectation. Conversely, a larger difference leads to a contrast effect, with perceived characteristics or preference shifting in the opposite direction of the expectation.

This dynamic interaction between expectation and perception highlights the crucial role of cognitive processes in shaping human responses. Such memories, experiences, and knowledge can be formed differently depending on the level of exposure to the food. Notably, as familiarity with the food increases through repeated exposure, the expectations become more specific (Ludden et al., 2009). Over time, these refined expectations contribute to a more stable and predictive framework for interpreting sensory stimuli.

**Fig. 1:**
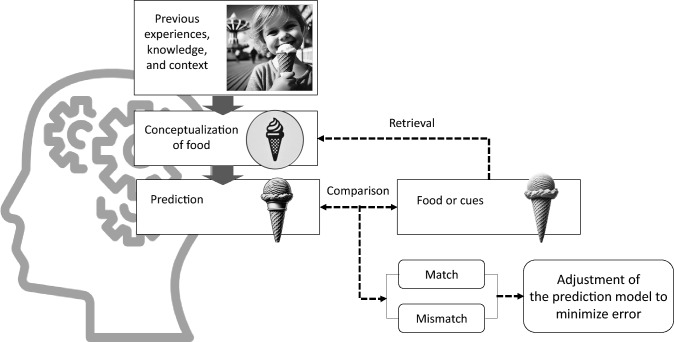
Predictive coding (Piqueras-Fiszman and Spence, 2015)

Building on this cognitive perspective, Piqueras-Fiszman and Spence (2015) proposed “predictive coding” as an explanation for how our liking is influenced by stored information (Fig. 1). Predictive coding posits that our brains interpret sensory information through a continuous cycle of hierarchical process involving predictions and error correction. Rather than passively reacting to sensory data, the brain generates predictions of lower-level sensory input based on higher-level models constructed using prior knowledge and context. For instance, encountering a label such as “gelato” serves as a cue that activates stored conceptualizations related to gelato, retrieving relevant knowledge and experiences such as flavors and textures. This retrieval generates prediction that are compared to incoming sensory information. If there is a match, our expectations are confirmed (assimilation), whereas mismatches prompt adjustment in our internal model to reconcile the differences (disconfirmation). This process illustrates how prior knowledge and experience shape our sensory perception and influence our overall hedonic responses.

However, when it comes to novel or unfamiliar foods for which no prior information has been accumulated, consumer test often yields ambiguous results that fail to clearly differentiate products based on liking. This can pose challenges for decision-making regarding product launches or improvement. Such issues are common in studies involving ethnic foods. For instance, in a study comparing the preference for bulgogi marinade between Korean and Japanese consumers (Park et al., 2017), the Japanese participants had difficulty clearly distinguishing between samples in terms of liking and familiarity, unlike Korean participants. Specifically, Japanese participants were unable to distinguish how close each sample was to the ‘authentic’ taste of Korea. This lack of differentiation likely stems from the Japanese participants’ limited exposure to bulgogi, which prevented them from establishing a clear sensory benchmark for what constitutes ‘authentic’ Korean flavor. Hong et al. (2011) reported that American consumers, who had less exposure to bulgogi, struggled to distinguish between varying levels of sweetness and toughness in different formulations of bulgogi. Additionally, their judgements of the ideal level of sensory attributes were less specific compared to Korean consumers who were more familiar with bulgogi. These findings underscore the role of sensory familiarity in shaping liking, highlighting the difficulties consumers face when evaluating foods that fall outside their habitual dietary experiences. Similarly, Peron and Allen (1988) reported that individuals who initially found it difficult to differentiate beer flavors improved their ability to do so through repeated exposure. This indicates that sensory learning, which occurs through repeated encounters with a food, can refine perception and ultimately influence liking. Therefore, it can be suggested that sensory perception also plays a key role in the development of liking for novel or unfamiliar foods.

Recent studies on acceptance, however, have primarily focused on how contextual factors, such as testing environment, consumption situations, and information such as price or labelling, affect consumer liking (Cardello and Meiselman, 2018). Research exploring the relationship between preference and sensory perception has mainly aimed at identifying the sensory drivers of liking to improve and optimize product formulations or has explored perceptual differences among individuals based on genetic factors, sociocultural background, and personal traits (Varela and Ares, 2018). While these studies provide valuable insights into the external and intrinsic factors influencing liking, they do not fully address how sensory perception itself evolves through exposure, nor how this evolution shapes consumer liking over time. In contrast, relatively few studies (Distel et al., 1999; Go et al., 2017; Jang et al., 2016; Park et al., 2020; ) have investigated the relationship between changes in sensory perception and the development of liking.

In today’s food market, novel products continue to emerge at a rapid pace. Innovative ingredients, such as alternative proteins and insect-based proteins, are driving the development of new markets (Andreani et al., 2023; Gravel and Doyen, 2020; Reyes-Jurado et al., 2021). Moreover, with the evolution of food culture, sensory perception has become increasingly important in shaping consumer interest in indulgent and gourmet foods like ethnic cuisine, wine, tea, beer, and artisanal products (Anderson, 2020; Biano, 2021). As consumer exposure to diverse food experiences expands, the interplay between sensory perception and liking becomes even more relevant, particularly in understanding how individuals adapt to new sensory stimuli over time. Given the growing segmentation of food consumption patterns, understanding consumers’ responses to unfamiliar sensory experiences and their liking for the novel foods is crucial to meeting the growing and diverse demands of the market.

Furthermore, as food acceptance not only influence one-time food choices but also have long-term impacts on dietary habits and health (Liem and Russell, 2019), it is important to further investigate how repeated exposure to certain foods and changes in sensory perception contribute to the formation of food acceptance. This line of inquiry is particularly significant in the context of promoting healthier eating habits, as increased familiarity with nutritious yet initially unfamiliar foods may facilitate their integration into regular diets. Such research is critical for advancing both the food industry and public health. This study, therefore, aims to review the current research findings on how changes in sensory perception during repeated exposure impact not only liking but also quality assessment and appreciation, while also suggesting potential directions for future research.

## How liking for novel foods is formed?

Food liking evolves dynamically with consumption experiences. In the study by Zandstra et al. (2000), which examined liking for bread with varying levels of saltiness over a week, the least liked bread was consumed the least on the first day. However, by the fifth day, the consumption of the bread initially perceived as least palatable equaled that of the bread originally considered most palatable. Additionally, previous studies (Drewnowski et al., 1982; Tuorila-Ollikainen et al., 1986; Vickers and Holton, 1998) have shown that while samples with stronger flavors or aromas were initially preferred, repeated consumption led to an increased preference for samples with milder flavors or aromas, sometimes even surpassing the initial favorites. Drewnowski et al. (1982) found that the soda with the strongest sweetness experienced the greatest decline in hedonic ratings after repeated exposure. Similarly, in the study by Tuorila-Ollikainen et al. (1986), white bread with optimal saltiness was most liked in the first evaluation, but after repeated consumption, bread with lower salt content became more favored. Vickers and Holton (1998) reported that although stronger iced tea was initially preferred over weaker iced tea, repeated exposure led to a shift in preference towards the weaker iced tea.

Such dynamic shifts in food liking align with well-established theories that explain how repeated exposure influences perception and preference. One of the most well-known mechanisms for the development of liking is Zajonc’s (1968) mere exposure theory. This theory posits that repeated exposure to a novel food reduces fear and uncertainty about its safety and taste, while increasing familiarity, and consequently, liking (Rozin and Fallon, 1986). This effect occurs without conscious awareness, meaning that active cognitive engagement with stimuli is not necessary to increase liking (Zajonc, 1968). The mere exposure effect does not always require a substantial number of exposures to influence liking (Havermans & van den Heuvel, 2023). There are instances where even a single exposure to a food can significantly increase its acceptance (Birch et al., 1998). Introducing new foods and dishes into one’s diet can encourage broader acceptance of a novel food through the “taste-like-chicken” effect, a phenomenon where familiarity is acquired by experiencing sensory similarities between new foods and already familiar foods (Havermans, 2009). This perspective highlights how food acceptance can develop passively over time, even without deliberate effort or conscious preference shifts.

Another prominent theory concerning changes in liking is associative learning, whose theoretical background is ground in classical and operant conditioning (Brunstrom, 2007; Rozin & Zellner, 1985). According to this theory, when a food is repeatedly paired with positive or negative experiences or stimuli, a preference or aversion for food is acquired based on these associations. Associative learning can take place in several forms. One example is flavor-nutrient learning, where a food is paired with positive nutritional outcomes like calorie intake. Another is flavor-consequence learning, in which positive post-ingestive effects, such as alertness or mood enhancement from caffeine or chocolate, influence liking. Lastly, flavor-flavor learning occurs when a food’s preference increases due to paring with already-liked sensory attributes or foods, such as sweetness (Havermans & van den Heuvel, 2023). Unlike mere exposure, which operates passively, associative learning suggests that repeated exposure influences liking through active reinforcement mechanisms, where the presence of physiological or hedonic rewards strengthens preference formation.

Additionally, foods with intense flavors, such as spicy foods, fermented foods, and those with a pronounced bitterness like coffee or tea, often have low initial acceptance when first encountered (Gotow et al., 2015; Go et al., 2017; Jang et al., 2016; Park et al., 2020; Xia et al., 2020) However, with repeated consumption, the liking for these foods tends to increase. From an evolutionary perspective, it is suggested that intense flavors are initially perceived as signals of potential danger, such as toxicity or spoilage caused by microorganisms, which triggers an aversive response. However, with repeated exposure, these foods with intense flavors are gradually recognized as safe and become associated with positive physiological responses, such as the release of endorphins (Ludy and Mattes, 2012; Rozin and Fallon, 1986; Rozin, 1990; Spence, 2018; Stevenson and Prescott, 1994; Stevenson and Yeomans, 1993). This shift in preference is thought to be influenced not only by cognitive changes, such as reframing, but also by perceptual changes over time. Although this assumption aligns with theoretical perspectives, empirical evidence directly supporting perceptual changes in response to repeated exposure remains limited.

While these mechanisms apply broadly to food preferences, their effects are particularly pronounced in the case of unfamiliar foods, where initial attitudes are more malleable. Compared to familiar foods that consumers’ preference has been already considerably stabilized, liking for unfamiliar foods is more likely to change as they become more exposed. Piqueras-Fiszman and Spence (2015) proposed that prior experiences and knowledge generate “situated conceptualization,” which is complex mental representations of food that simulate expected sensory and contextual details. These representations are retrieved when we encounter food, enabling the brain to interpret sensory information. Song et al. (2019) found that when a stir-fried spinach dish was made using Korean doenjang, a condiment unfamiliar to Indonesian consumers, they initially could not differentiate between the samples in terms of liking. However, after four repeated exposures, they were able to identify which doenjang best complemented the spinach dish to their taste. This result suggests that repeated exposures developed a frame of reference for the most preferred sensory profile (Chung, 2019). Thus repeated exposure seems to refine sensory discrimination, ultimately leading to a more stable and informed preference.

## The relationship between perceptual changes and development of liking

In many cases, repeated exposure not only changes acceptance but also alter sensory perception by either increasing sensitivity or inducing adaptation. Simply being repeatedly exposed to certain foods can enhance the ability to detect specific sensory attributes in food. As familiarity with an aroma increases, it is perceived as more intense, and there is a positive correlation between the perceived intensity of the aroma and pleasantness (Distel et al., 1999). Gotow et al. (2015) reported that frequent consumers of canned coffee beverages rate bitterness and retronasal aroma more intensely than less frequent consumers. They suggested that frequent consumption in daily life enhances familiarity with coffee, which in turn increases sensitivity to its afterflavor. Similarly, a study by Xia et al. (2020) comparing the results of Check-All-That-Apply (CATA) tests between heavy users and light consumers of chrysanthemum tea and coffee beverages found that the heavy user group identified significantly more CATA terms that distinguished between samples, especially for complex characteristics such as ‘full-bodied’ compared to the light user group. This suggests that as individuals become more experienced with a particular food, their sensory system becomes more attuned to detecting nuanced attributes, potentially reinforcing their preference for the food. This has been related to perceptual fluency (McKean et al., 2020; Reber et al., 1998), which refers to the subjective feeling of ease or difficulty when processing sensory information.

While repeated exposure can enhance sensitivity, it can also lead to adaptation, where individuals become less responsive to certain sensory attributes over time. Previous studies (Engel et al., 2006; Tanimura and Matters, 1993) have reported that non-user or infrequent consumers of a particular food are more sensitive to its bitterness or pungency. Also, Xia et al. (2020) found that frequent consumers are less sensitive to negative attributes of coffee, such as astringency and earthy flavors, compared to infrequent consumers. Masi et al. (2015) noted that infrequent coffee consumers were more sensitive to negative attributes like earthy flavors. Go et al. (2017) observed that in a CATA test of various natural cheeses, Korean consumers who were initially unfamiliar with cheeses such as Emmental, Gorgonzola, and Parmigiano Reggiano, perceived strong negative sensory characteristics such as bitterness, off-flavors, and a lack of balance. However, after repeated exposure, the frequency of selecting these negative terms decreased.

This shift suggests that perceptual changes play a key role in transforming an initially aversive or unfamiliar sensory experience into a more favorable one, thereby facilitating an increase in liking over time. Notably, the preference for foods with strong flavors, as mentioned earlier, can be influenced not only by their association with positive post-ingestive responses but also by changes in sensory perception. In the case of American consumers, those with more experience consuming kimchi tend to prefer spicier and more fermented kimchi over milder and less fermented kimchi. This preference can be explained by their adaptation to the spicy sensation and strong fermented flavors, which enhances the processing of other sensory information (Jang et al., 2016; Park et al., 2020). It has been suggested that repeated exposure to strong flavors, such as those from fermentation or spiciness, leads to adaptation, reducing initial aversion and allowing consumers to better process sensory information, thereby enabling the recognition of various flavors that were not noticed on the first trial (Köster et al., 2003). Lévy et al. (2006) and Berlyne (1967) reported that when individuals are exposed to samples with a level of complexity exceeding the optimal level for maximizing liking, their liking tends to shift towards more complex samples. In the context of food, complexity is understood as the variety and number of perceived sensory attributes and the extent to which they can be distinguished from each other (Palczak et al., 2019). Therefore, the increased liking for well-aged fermented foods, such as kimchi or cheese, is likely driven by an inclination toward more complex flavors that become more recognizable after repeated exposure. This implies that perceptual changes not only increase sensitivity to individual sensory attributes but also foster an appreciation for more elaborate and multifaceted flavor compositions.

## Perceptual learning

The increase in familiarity and the corresponding changes in perception due to repeated exposure are thought to be related to perceptual learning. Perceptual learning refers to long-term changes in perceptual abilities resulting from experience or repeated practice (Connolly, 2017; Goldstone, 1998). In other words, experience enables the perception of characteristics that were previously unnoticeable (acquired perception, Reid, 1997), and these characteristics become part of the contents of perception used to understand objects and environments. For instance, if someone were to cut only pine trees in a forest for several months, they would become adept at recognizing the key characteristics of pine trees and distinguishing them from other types of trees (Siegel, 2011). This acquired perception may involve high-level experience properties of being a pine tree, or low-level properties such as color, shape, texture, or sound (Connolly, 2017; Siegel, 2006, 2011).

Goldstone (1998) identified four types of perceptual learning: differentiation, unitization, attentional weighing, and stimulus imprinting. Differentiation involves recognizing differences in stimuli that were previously undetectable. Through differentiation, stimuli that were once psychologically merged and indistinguishable become separated and identified as distinct percepts. Through this process, perceptual learning allows for finer distinctions in sensory input, ultimately enhancing discrimination between similar stimuli. Unitization, in contrast to differentiation, simplifies tasks by grouping multiple features of complex stimuli into a single unit (“chunking”). This process means that features previously perceived as separate are now integrated into a unified whole, forming a new property. By integrating multiple features into a single perceptual entity, unitization reduces cognitive load and facilitates more efficient information processing. Attentional weighing focuses attention on distinguishing features of stimuli, while less salient features are less clearly differentiated. Through attentional weighing, repeated practices or experiences lead to automatic attention to important dimensions or characteristics. In this way, perceptual learning optimizes cognitive resources by prioritizing the most relevant aspects of sensory input. This mechanism is related to categorical perception, where stimuli from the same category are perceived more distinctly than stimuli from different categories. Stimulus imprinting refers to the development of specialized detectors or receptors in our perceptual system for stimuli that are repeatedly encountered. With increased exposure, detection becomes quicker, more accurate, and information processing becomes smoother. In essence, stimulus imprinting helps in perceiving stimuli that are ambiguous or briefly exposed. Together, these mechanisms illustrate the diverse ways in which perceptual learning enhances sensory processing and cognition.

Attention is an important factor that influences perceptual learning (Gold and Watanabe, 2010). Attention to a specific task or relevant sensory features seems to be crucial for initiating certain types of perceptual learning. Notably, the role of attention in perceptual learning suggests that experience alone is not always sufficient; rather, how stimuli are attended to influence the extent and nature of learning. Tsushima and Watanabe (2009) discussed the role of top-down attention, bottom-up attention, or mere exposure without any attention in perceptual learning. Top-down attention involves cognitive strategies that voluntarily focus attention on important features associated with the task while filtering out task-irrelevant features. In contrast, bottom-up attention is involuntarily drawn to stimuli that stand out due to the presence of salient features (Desimone and Duncan, 1995; Moran and Desimone, 1985). Under some conditions, perceptual learning could result from mere exposure alone, without involving either top-down or bottom-up attention. Since this effect does not rely on attention, they inferred that perceptual learning can take place for features that do not receive focused attention (Gold and Watanabe, 2010). These findings highlight the complexity of perceptual learning, demonstrating that different types of attention, or even the lack thereof, can shape how individuals process and internalize sensory information.

Perceptual learning of flavors has mainly been investigated in animal studies. Previous studies (Bennett and MacKintosh, 1999; Mondragón et al., 2002; Rodriguez and Alonso, 2004; Recio et al., 2016; Sánchez et al., 2022; Symonds et al., 2002) have reported that exposing rats to two types of stimuli in advance enhanced their ability to discriminate between these stimuli. This aligns with broader theories of perceptual learning, which suggest that repeated exposure leads to increased sensitivity to distinguishing features of stimuli. In particular, alternating preexposure, where subjects are exposed to different stimuli in an alternating manner, led to better discrimination compared to blocked preexposure, where subjects are exposed to the same stimuli in consecutive blocks. This suggests that alternating preexposure helps in detecting distinctive features and reduces generalization, indicating that perceptual learning benefits from variability in exposure. Enhanced discrimination ability through alternating pre-exposure has also been confirmed in humans (Dwyer et al., 2004), supporting the notion that perceptual learning operates similarly across species. Dwyer et al. (2004) proposed that associative learning might play a significant role in perceptual learning, suggesting that alternating preexposure created associations that inhibit generalization between the two stimuli. These studies indicate that perceptual learning can influence flavor aversion and preference in the context of associative learning. Flavor aversion occurs when a specific flavor is associated with a negative consequence, and preference is known to generalize across similar flavors. Decreased generalization reduced the ability to associate other similar flavors with negative consequences and limits the transfer of learned responses from one flavor to another, allowing for the development of more specific aversion or preference (Symonds et al., 2002; Mondragóon et al., 2002; Dwyer et al., 2004; Sergio et al., 2016).

## Perceptual learning in food

Repeated exposure to food can induce perceptual learning (Reid, 1997), which helps in constructing the sensory representation of the sample by utilizing the sensory characteristics acquired through these exposures. This repeated exposure, and the resulting increase in familiarity, can enhance the ability to distinguish between different sensory attributes by focusing attention on distinctive features (Bende and Nordin, 1997). This process corresponds to attentional weighing in perceptual learning. Additionally, Spence (2019) suggested that attention is related to the mechanisms of differentiation and unitization within perceptual learning. Thus, as individuals repeatedly encounter specific food stimuli, their perceptual system enhances its ability to process and categorize these sensory inputs, resulting in a more structured and efficient recognition of flavors and aromas. Furthermore, familiar stimuli lead to more refined perceptual representations, enabling a clearer distinction between the perceptual boundaries of different stimuli (Rabin, 1988).

Perceptual learning in food systems is often investigated by comparing the detection, identification, and discrimination abilities of expert and non-experts in the wine industry (Connolly, 2017; Spence, 2019; Spence and Wang, 2019; Tempere et al., 2019; Table 1). The detection thresholds for aromas in experts are generally not significantly different from those in non-experts. Specifically, for non-wine-related aromas like 1-butanol (which has a chemical-like scent), there is no notable difference in detection threshold levels between experts and non-experts (Bende and Nordin, 1997; Parr et al., 2002, 2004). Pickering et al. (2007) also found no significant difference in detection threshold of 2-isopropyl-3-methoxypyrazine (off-odor associated with wine defects) between winemakers and non-winemakers.

**Table 1:** Summary of research outcomes comparing perceptual abilities between wine experts and novices

Tasks	Stimuli	Subjects	Assessment methods	Results	References
Detection	1-butanol solutions	Wine experts (n = 16) and novices (n = 16)	2-AFC^1^ test	No significant difference between experts and novices	Bende and Nordin, (1997)
	1-butanol solutions	Wine experts (n = 11) and novices (n = 11)	2-AFC test	No significant difference between experts and novices	Parr et al. (2002)
	1-butanol solutions	Wine experts (n = 14) and novices (n = 14)	2-AFC test	No significant difference between experts and novices	Parr et al. (2004)
	Phenylethyl alcohol (Sniffin’ stick)	Sommelier students (n = 25) and novices (n = 29)	Triangle test	No significant difference between trainees and novices	Poupon et al. (2019)
	2-isopropyl-3-methoxypyrazine (off-odor) in red and white wines	Winemakers (n = 9) and non-winemakers (n = 14)	Triangle test of orthonasal and retronasal odor stimuli	No significant difference between winemakers and non-winemakers	Pickering et al. (2007)
Discrimination	Citral and eugenol in mineral oil	Wine experts (n = 22) and novices (n = 22)	Discriminating citral from eugenol	Experts outperformed novices	Bende and Nordin, (1997)
	32 odors (Sniffin’ stick)	Sommelier students (n = 25) and novices (n = 29)	Triangle test	No significant difference between trainees and novices	Poupon et al. (2019)
Identification	16 common household odors (floral, woody, fruity, pungent, etc.)	Wine experts (n = 22) and novices (n = 22)	Multiple-choice test to identify target odorants from a list of 4 options	Experts outperformed novies for a few odors	Bende and Nordin, (1997)
	24 wine-related odors	Wine experts (n = 11) and novices (n = 11)	Giving a verdict on whether the odor was previously presented or not	No significant difference between experts and novices	Parr et al. (2002)
	27 wine-related odors	Wine experts (n = 14) and novices (n = 14)	Giving a verdict on whether the odor was previously presented or not	No significant difference between experts and novices	Parr et al. (2004)
	16 odors (Sniffin’ stick)	Sommelier students (n = 25) and novices (n = 29)	Multiple-choice test to identify target odorants from a list of 4 options	Trainees outperformed novices in identifying target odors	Poupon et al. (2019)
	47 wine-related or non-wine related odors	Wine experts (n = 39) and novices (n = 41)	5-AFC^1^ test	Experts were more accurate in olfactory identification	Tempere et al. (2016)
	50 odors including 10 wine-related odors, as well as 41 red and white wines	Untrained wine drinkers, second- and third-level trainee sommeliers, and professional sommeliers, (n = 12 per group)	Identifying the matching names for wine-relevant and common odors	Professionals outperformed trainees in identification of wine-relevant odors, not common odors	Zucco et al. (2011)
Recognition	24 wine-related odors	Wine experts (n = 11) and novices (n = 11)	5-point confidence rating for identification of the name of odorants	Experts showed superior recognition of olfactory stimuli	Parr et al. (2002)
	27 wine-related odors	Wine experts (n = 14) and novices (n = 14)	5-point confidence rating for identification of the name of odorants	Experts showed superior recognition of olfactory stimuli	Parr et al. (2004)
	50 odors including 10 wine-related odors and 41 red and white wines	Untrained wine drinkers, second- and third-level trainee sommeliers, and professional sommeliers, (n = 12 per group)	Multiple choice test of a target wine-relevant odor, and common odor, and a target wine from a set of 4 odors	Both professional and trainee sommeliers performed better in wine recognition, but the level of training did not significantly affect the recognition of common and wine-relevant odors	Zucco et al. (2011)

^1^Alternative forced choice test

Unlike the findings from the detection threshold, there are more studies supporting experts’ superior abilities in odor discrimination and identification compared to novices (Parr et al., 2002, 2004; Zucco et al., 2011). Tempere et al. (2016) reported that wine experts are more adept at identifying everyday odors and those related to wine defects than novices. Similarly, Poupon et al. (2019) found that students in sommelier training programs, who receive specialized education in wine, possess better odor identification abilities compared to untrained students. Bende and Nordin (1997) also reported that experts could detect the addition of citral (lemon odor) added to eugenol (clove odor) at concentrations below those detectable by non-experts. Moreover, they found that the experts were more proficient than the general public at identifying lemon, orange, cinnamon, and lilac odors. However, experts’ superior performance in identification was observed only for a subset of the household aromas, rather than across the entire olfactory spectrum. These findings highlight the need for further studies to clarify the extent to which perceptual learning, acquired through sensory experiences, influences the discrimination and identification of odors in food.

## Effect of intentional learning on perception acquisition

Intentional learning, such as sensory training or tasting classes, can affect perception by inducing perceptual learning (Table 2). Persistent changes are a key criterion of perceptual learning, but they have not been fully confirmed. Rabin (1988) reported that certain training methods, such as labeling an odor or using an attribute list for sensory profiling of target orders, significantly improved odor identification ability. However, such enhancement was not observed in the identification of untrained odors, indicating no transfer of learning to other odors. These stimulus-specific effects of the intentional training raise questions about its broader applicability to untrained sensory inputs.

**Table 2:** Summary of research outcomes investigating changes in perceptual abilities by intentional training

Task	Stimuli		Subjects		Training	Assessment methods		Results	References
Detection	Benzaldehyde and 5-methylfurfural (cherry-like), and isoamyl acetate (banana-like)		8 – 12 subjects		Repeated threshold testing	2-AFC^1^ test		Repeated testing decreased sensitivity to benzaldehyde and methylfurfural in female subjects, but not to isoamyl acetate	Dalton et al. (2002)
	A beer sample supplemented with isoamyl acetate		10–20 subjects		15 h of training to detect added aroma and to evaluate the intensity of general components	Triangle test before and after training		Training lowered detection threshold for the aroma	Chollet and Valentin, (2006)
	Diacetyl and linalool		2 groups, 16 wine experts per group (N = 32)		Sniffing diacetyl or linalool 2–3 times daily for 1 min over the course of 1 month	3-AFC test		Training reduced the detection threshold for the trained aroma, but not for the untrained aroma	Tempere et al. (2012)
	n-butanol (Sniffin’ stick)		14 subjects that received training and 12 untrained subjects		5-week blind wine tasting training (on average more than once per week)	Triangle test before and after training		Olfactory threshold of both trained and untrained subjects decreased in the second test	Wang et al. (2021)
Discrimination	Amyl butyrate, butyl alcohol, cinnamyl n-butyrate, octanal, phenethyl alcohol, propyl butyrate, and trans-2-decen-1-al		4 groups, 14 subjects per group (N = 56)		No training (control), label training on targets, profiling task, and label training on non-targets	Two-interval, same-difference task		Label training on the target and profiling task most significantly enhanced discrimination abilities	Rabin, (1988)
	9 brands of bottled beer		4 groups, 10 wine novices per group (N = 40)		Control, verbal training, taste training, verbal/taste training	Triangle test, similarity rating		Taste experience, not experience with beer-flavor terminology, improved novices’ discrimination ability	Peron and Allen, (1988)
	10 similar pairs and 10 dissimilar pairs of commercial beers		16 subjects		15 h of training to detect added aroma and to evaluate the intensity of general components	Same-difference test		The short training does not affect discrimination abilities	Chollet and Valentin, (2006)
	12 pairs of learned commercial beers, 12 pairs of learned beers with added aroma, and 12 new beers with added aroma		10 subjects trained for 72 h and 10 untrained subjects		72 h of training to detect added aroma and to evaluate intensity of general components	Same-difference test		Trained subjects outperformed untrained subjects in discrimination of learned beers, but not new beers	Chollet and Valentin, (2006)
	32 odors (Sniffin’ stick)		14 subjects that received training and 12 untrained subjects		5-week blind wine tasting training (on average more than once per week)	Triangle test		While the training group’s performance improved over time, the control group’s performance declined	Wang et al. (2021)
	30 lager beers		16 subjects		0, 8 24, and 32 h of training to detect added aroma and to evaluate intensity of general components	Finding the name of aroma added in the beer among a list of descriptors		Training increased the percentage of correct identification, but the percentage of correct rejection decreased	Chollet and Valentin, (2006)
	104 white wines and 109 red wines		15 trained wine tasters, 9 novices, and 6 experienced tasters		18 blind wine training sessions for 5 weeks	Writing tasting notes and identifying grape variety, vintage, and place of origin		Blind tasting training improve accuracy of guess about the variety and acidity	Wang and Prešern, (2019)
	16 odors (Sniffin’ stick)		14 subjects who received training and 12 subjects with no training		5-week blind wine tasting training (on average more than once per week)	A multiple-choice test to identify target odorants from a list of 4 options		The training had no significant effect on the recognition ability in either group	Wang et al. (2021)
Recognition	8 red wines		20 intermediate wine drinkers (with over 5 years of drinking experience) and 20 novices		Writing description of the target wine or solving a puzzle after tasting it	Identifying the target from among 4 samples and rating their confidence in their choice on a 7-point scale		Both novices and intermediate drinkers performed better in the description writing condition than the control condition	Hughson and Boakes, (2009)

^1^Alternative forced choice test

Previous studies have reported a significant increase in sensitivity due to training or repeated testing over time (Chollet and Valentin, 2006; Dalton et al., 2002; Tempere et al., 2012) (Table 2). In these studies, detection threshold decreased in a stimulus-specific manner. Chollet and Valentin (2006) observed that after 15 h of training in detecting and identifying isoamyl acetate, the detection threshold for the compound in beer tended to decrease. Dalton et al. (2002) found that repeated threshold testing of benzaldehyde over 10 trials slightly decreased the detection threshold of 5-methylfurfural (a cherry-like odor similar to benzaldehyde), but did not affect the threshold for amyl acetate (an apple/banana-like odor, perceptually dissimilar to benzaldehyde) in female subjects. Tempere et al. (2012) observed a significant decrease in the detection thresholds for a trained stimulus after a month of training, but no such change was found for the untrained stimulus. These results suggest that the effect of intentional learning, at least in the context of detection sensitivity, is highly stimulus-specific and does not necessarily extend to perceptually distinct stimuli. However, whether the increased sensitivity due to training persists long-term still remains to be confirmed in future studies.

Studies have consistently reported that participants’ ability to differentiate and identify samples improved with training (Rabin, 1988; Wang and Prešern, 2019) (Table 2). Peron and Allen (1988) found that providing additional taste experience to assessors who initially struggled to distinguish between seven types of beer improved their ability to differentiate them. Similarly, Chollet and Valentin (2006) reported that training increased the ability to identify aroma components added to beer from 25 to 45%. After five weeks of blind tasting training, participants demonstrated improved inference of the grape varieties used in wines, which can be seen as a higher form of perceptual learning related to categorization (Wang and Prešern, 2019; Spence and Wang, 2019). Tempere et al. (2019) proposed that the enhanced ability to distinguish samples might result from increased sensitivity to weak and ambiguous stimuli, and decreased sensitivity to irrelevant features, both of which occur with repeated exposure. These changes in sensitivities allow individuals to selectively focus on relevant and important sensory cues against the background.

Meanwhile, some findings challenge the notion that the effect of intentional learning on perception acquisition is strictly stimulus-specific, raising the possibility that certain training regimens may facilitate broader generalization. Wang et al. (2021) found that the group undergoing 5 weeks of blind wine tasting training had a significantly higher discriminating ability compared to the control group. Notably, this effect was particularly pronounced after participating in ten or more training sessions. Moreover, the enhanced sensory discrimination abilities were related to odors not specifically used during the training, suggesting that blind wine tasting training might generalize to olfactory discrimination performance with other odors, including non-wine-related-aromas. The previous finding by Bende and Nordin (1997), which showed that wine professionals were better at distinguishing citral from clove, also supports that training effects are generalized to other odors. They proposed that professional wine tasters have relatively limited exposure to lemon and clove odors in their profession, and thus, the observed result might result from the generalization of perceptual learning to other odors. However, they concluded that this generalization could be attributed to cognitive strategies for odor discrimination, rather than an increased general interest in odors that leads to greater attention to their distinguishing features. However, these results contradict previous studies that suggest sensitivity increased only to trained odors, not untrained ones (Dalton, 2002; Tempere et al., 2012). In addition, subjects who underwent 72 h of training in detecting and identifying aromas added to beer were better at discriminating beers containing the trained aroma than they were at distinguishing new beers (Chollet and Valentin, 2006). These inconsistencies in the findings underscore the need for further research to determine whether the effects of training can be transferred to untrained stimuli.

It should be noted that repeated exposure can enhance discrimination even without the need for explicit learning (Hughson and Boakes, 2009; Owen and Machamer, 1979). When participants were asked to differentiate a target odor from a mixture of the target and a contaminant odor, both the familiar target and contaminant were more salient and detectable than unfamiliar odors (Rabin, 1988). Houghson and Boakes (2009) proposed that casual, untutored experience can enhance wine recognition. Their findings showed that both experienced wine drinkers (with an average of 9 years of wine-drinking experience) and novices (those who had an average of 1.25 years of experience) performed significantly better in a wine recognition task when they were asked to describe the target wine prior to the test, as opposed to solving a crossword puzzle. These findings indicate that passive perceptual learning, gained from the experiences naturally accumulated through repeated exposure in our daily life, can improve odor discrimination.

Overall, the effects of intentional learning and untutored experience on perceptual enhancement in food contexts may be closely related to the role of attention in perceptual learning, as discussed earlier. Specifically, intentional learning may facilitate top-down attention by directing cognitive focus toward relevant sensory attributes and reinforcing perceptual discrimination through structured training. In contrast, perceptual learning through mere exposure during everyday encounters with foods occurs organically, through unconscious and automatic mechanisms. Repeated exposures in daily life increase familiarity, which may in turn enhance attention to specific sensory features while filtering out irrelevant stimuli, thereby promoting bottom-up attentional processes. However, the underlying mechanisms through which perceptual learning is induced, whether through intentional learning or everyday exposure, remain largely unexplored. To the best of our knowledge, however, no studies have systematically investigated how intentional learning modulates perceptual learning through attentional mechanisms. It is also unclear whether this modulation leads to long-term changes in perceptual ability, which is a defining characteristic of perceptual learning, particularly in the context of food. Future research should seek to provide empirical evidence clarifying the distinct contribution of intentional learning and passive exposure in shaping perceptual learning, as well as the attentional mechanisms that mediate these effects.

Beyond attention, memory process may also play a crucial role in perceptual learning. It has been suggested that working memory and long-term memory interact to facilitate perceptual learning by enhancing the encoding retrieval, and processing of perceptual information (Bartsch et al., 2024; Gong et al., 2023; Hirschstein et al., 2022; Megla and Bainbridge, 2024). Working memory, which serves as a temporary storage system, prioritizes and manipulates sensory information, thereby strengthening long-term memory representations. Long-term memory, in turn, provides a repository of past experiences that can influence current perceptual processes. In a study on visual perception, Stokes et al. (2012) used fMRI and EEG measurements to confirm that attentional tuning cued by past experience sharpens perceptual sensitivity for target stimuli. This suggests that prior knowledge stored in LTM continuously fine-tunes sensory processing, thereby improving perceptual abilities.

Long-term memory consists of two primary subtypes, explicit and implicit memory (Dayan and Guillery-Girard, 2010). Implicit memory involves perceptional and emotional memories that are recalled unconsciously and can prime behaviors, thereby improving perceptual performance without intentional retrieval. In contrast, explicit memory requires conscious effort to recall previously learned information. The roles of explicit and implicit memory in perceptual learning remain insufficiently explored in the food domain. Given the unconscious nature of both implicit memory and perceptual learning, it is plausible that implicit memory may be primarily engaged in perceptual learning processes. However, in intentional learning scenarios, explicit memory, particularly the retention of structured information acquired through training or formal instruction may contribute to perceptual learning to a greater extent than in passive, daily experiences. While the role of explicit memory in perceptual learning within the food context remains largely unexplored, insights can be drawn from research on visuo-perceptual learning. For example, Fama et al. (2004) examined visuo-perceptual learning in non-amnesic alcoholics with impaired explicit memory compared to a control group. Despite deficits in explicit memory, alcoholics were able to perform visuo-perceptual learning tasks at levels comparable to controls. This suggests that they relied on alternative cognitive strategies, which, while effective, were more cognitively demanding and less efficient. These findings imply that while explicit memory is not essential for perceptual learning, it enhances the efficiency of the learning processes. Future studies should further investigate how explicit and implicit memory contribute to perceptual learning in food contexts, particularly in relation to intentional learning versus passive exposure. Understanding these mechanisms could provide valuable insights into optimizing training approaches for sensory expertise and improving consumer acceptance of novel or health-promoting foods.

## Interplay between perceptual learning and cognitive processing

In the case of intentional learning, the extent to which it facilitates perceptual learning depends on the content of the learning program. If the program includes information about the relevant stimuli, such as their characteristics and attributes, it may engage cognitive processing that supports and enhances perceptual learning. By providing structured knowledge, this cognitive engagement can reinforce the recognition and differentiation of sensory attributes, thereby promoting more refined perceptual learning. Previous studies (Houghson and Boakes, 2001; Spence, 2019) reported that perceptual learning in food can be influenced not only by perceptual input but also by cognitive top-down processing. Individuals with a higher level of wine knowledge, including academic degrees in enology, demonstrated lower detection thresholds for odors caused by wine defects such as diacetyl and ethylphenols (Tempere et al., 2011; Schumaker et al., 2017). It has been suggested that the superior identification and recognition abilities of experts arise from their domain-specific conceptual knowledge. While experts categorize wines based on their varietal origins, non-experts tend to classify wines by perceptual features (e.g., fruity) or dimensions (e.g., sweet) since knowledge of wine characteristics related to grape varieties influences the perception of the wine (Solomon, 1997).

It appears that cognitive input shapes the way individuals store, retrieve, and apply sensory information during tasting experience. Cognitive training on background information such as wine-growing regions, production methods, grape varieties, history, and descriptors helps less experienced wine consumers create a more robust memory of their taste experiences, which in turn improves their performance on wine recognition tasks (Latour et al., 2011). Urdapilleta et al. (2011) found that less experienced wine evaluators exhibited considerable individual differences in judging the typicality of wines based on region and variety, suggesting that they do not share the same cognitive constructs related to wine. In contrast, experts’ judgments were relatively homogeneous. They propose that wine experts employ domain-specific knowledge in their top-down processes when determining similarities. Additionally, experts used cognitive strategies that compare wines against exemplar representations when assessing typicality. These prototypical representations are developed through higher-order cognition involved in top-down information processing (Parr, 2019). These findings highlight the interplay between perceptual learning and conceptual framework, reinforcing the idea that top-down processes improve sensory discrimination through repeated exposure and cognitive engagement.

Tempere (2010) found that the rejection threshold for earthy odor in wine was reduced when participants were trained with geosmin reference samples and instructed to focus on earthy odors. In contrast, when no reference samples or instructions were given, there was no change in the rejection threshold. This suggests that perceptual training combined with associative learning where information about stimuli is provided can impact perceptual abilities. As previously mentioned, this type of associative learning seems to facilitate perceptual learning by enhancing attention to more relevant perceptual features and dimensions, while reducing attention to irrelevant features, through attentional weighing (Tempere et al., 2019).

Previous neuroimaging studies also supported the involvement of cognitive aspect in enhanced perceptual performances (Tempere et al., 2019). Wine experts, such as sommeliers, showed increased activity in the left insula and orbitofrontal cortex in the brain that are involved in high-level cognitive processes, such as decision making. In contrast, non-experts activated the primary gustatory cortex and the regions that process emotions (Castriota-Scanderbeg et al., 2005; Li et al., 2006; Pazart et al., 2014). In addition, master sommeliers demonstrated a larger volume of grey matter in the endothrinal cortex whose function is being a memory network hub compared with controls (Banks et al., 2016). Sreenivasan et al. (2017) found that sommeliers exhibited increased connectivity in the network of brain regions involving higher-level cognitive processes. Based on these findings, it is speculated that expertise in sensory domain is not merely a function of repeated exposure but also the result of deep cognitive restructuring and neural adaptation, which reinforces the argument that perceptual learning is tightly coupled with high-order cognition.

Furthermore, the role of cognitive processing in perception acquisition is prominently highlighted in semantic learning and training related to sensory attributes. Semantic training helps assessors develop common sensory terms and concepts (Tempere et al., 2019), while also enhancing memory and identification abilities related to smell (Clapperton and Piggott, 1979; Gawel, 1997; LaTour and Deighton, 2019; Peron and Allen, 1988; Rabin, 1988; Tempere et al., 2014; Walk, 1966; Wang and Prešern, 2019). Hughson and Boakes (2009) found that subjects who verbally described the sensory characteristics of wine performed better in wine recognition tasks. It can be argued that verbalization not only strengthens perceptual encoding but also facilitates the formation of stable cognitive categories, thereby making it easier to retrieve and apply sensory knowledge in future evaluations. Describing a wine sample prompts more focused and sustained attention to its features, leading to more effective perceptual encoding. Rabin (1988) reported that novices trained to label specific odors showed a significant improvement in their ability to distinguish these odors after just one day. This finding is consistent with previous studies suggesting that verbalization or labeling of stimuli improves identification abilities (Melcher and Schooler, 1996; Solomon, 1990). These results suggest that providing names and information about sensory attributes through training, and reinforcing this knowledge through experience, likely clarifies the boundaries of sensory attribute categories, thereby enhancing the ability to differentiate features and strengthening categorical perception (Rabin, 1988; Livermore and Laing, 1996).

## Perceptual learning in appreciation and quality perception

Repeated exposure can alter discrimination ability or preferences, which in turn can influence quality perception, appreciation, and, consequently, food choice. High-involvement consumers often perceived more complex and diverse sensory characteristics than novices or low-involvement consumers, which enables them to make better judgement about ‘good’ and ‘bad’ products. In fact, in the case of wine, consumers with more experience or knowledge are reported to recognize and differentiate the quality of wine better than novices, as they can identify various subcomponents and distinguishable dimensions of quality (Charter and Pettigrew, 2007; Jover et al., 2004; Torri et al., 2013).

The dictionary definition of appreciation is “the recognition and enjoyment of the good qualities of someone or something” (Oxford Languages, 2022) or “the recognition of aesthetic values” (Merriam-Webster, 2022). Appreciation involves the pleasure derived from a stimulus, the interaction between sensory, emotional, and cognitive responses, personal preferences, and training or the evaluation procedures that often require intensive attention (Charters and Pettigrew, 2005). Although studies on food appreciation is limited, existing studies indicate that aesthetic appreciation is closely linked to perceptual learning, where the brain’s ability to detect and respond to sensory stimuli is refined through exposure and practice. For example, Sarraso et al.(2022) found that aesthetic appreciation of musical intervals correlates with larger mismatch negativity responses in the brain, which serve as markers of perceptual learning. This suggests that the appreciation of music is enhanced as the brain becomes more adept at recognizing and processing musical patterns. For the visual stimuli, more appreciated abstract stimuli were associated with perceptual processing fluency such as faster response times and enhanced attentional engagement (Sarrasso et al., 2020).

When perceptual learning occurs during the appreciation process, it updates the mental representation of the sensory environment. As sensory uncertainty about the environment decreases and new information is acquired, the brain generates an intrinsic self-generated reward, which in turn triggers aesthetic pleasure. Subjective experiences, such as expertise, along with contextual and cultural factors, have been suggested to influence appreciation by modulating judgments about specific features that signal a more reliable informational gain (Sarraso et al., 2022). In the case of wine, general wine consumers tend to form mental representations of wine samples based on their liking, while wine experts do so based on grape varieties or overall quality (Honore-Chedozeau et al., 2019). Similarly, Korean consumers familiar with green tea develop mental representations based on sensory differences related to the origin and processing methods of the tea, whereas French consumers unfamiliar with green tea base their representations on personal preference (Kim et al., 2013). Tempere et al. (2019) suggested that as individuals gain more experiences with wine, their mental standards or prototypes of a wine’s sensory characteristics become more refined. Thamke et al. (2009) proposed that sensory frames are constructed using easily perceived characteristics such as sweetness, sourness, and bitterness, and with sensory attributes fitting these frames being drawn from memory and actual sensory experiences. Therefore, it can be concluded that perceptual learning through repeated exposures likely plays a role in forming sensory frames for appreciation by influencing memory and actual sensory experiences related to sensory attributes.

Similar to appreciation, perceptual learning may impact how individuals interpret and assess quality by enhancing sensitively to sensory cues, based on their evolving perceptual experiences. The quality of a product can be defined differently depending on the perspective from which it is viewed, such as whether it is technical, product-oriented, or consumer-oriented (Steenkamp, 1990). Fundamentally, quality is a multidimensional and abstract concept that encompasses various elements necessary to satisfy consumer expectations and needs (Jover et al., 2004; Peri, 2006). Since the judgment of quality varies according to the consumer’s purpose, needs, and perceptions, the term ‘perceived quality’ is often used instead of simply ‘quality’ (Garvin, 1984; Steenkamp, 1989). Steenkamp (1989) stated that perceived quality, which includes liking, is based on the perception of product characteristics. Consumers predict quality before consumption using various quality cues (expected quality), and judge it based on their experience of quality attributes during actual consumption (experienced quality) (Steenkamp, 1989). From this concept of quality, two key points should be noted. First, despite the multidimensional nature of the concept of the quality, the quality of food is closely linked to sensory perception. Second, it becomes evident that the evaluation of quality varies depending on the individual, the context, and the specific cues and attributes used for judgment (Holm and Kildevang, 1996). Therefore, the perception and evaluation of quality can be understood as being shaped by how an individual perceive specific attributes and which factors influence this perception.

**Fig. 2:**
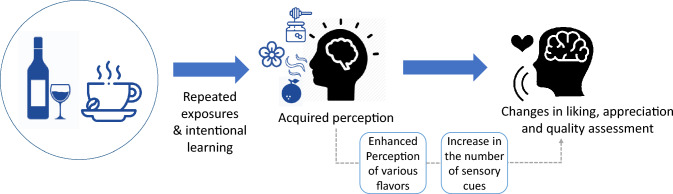
The proposed role of perceptual learning in development of liking, appreciation, and quality assessment

It is thought that, as perceptual learning enhances the perception of various sensory attributes, the increase in the number of quality cues and attributes that consumers can use during quality evaluation is likely to influence the judgment of quality (Fig. 2). This influence is expected to be particularly significant in the case of foods with complex flavors, such as coffee, tea, and wine. For wine, factors such as the harmony of flavors, the complexity of aromas, and the intensity and balance of aromas generated during fermentation and aging, known as the bouquet, are some of the key quality indicators (Jover et al., 2004). The perception of these related sensory attributes would greatly contribute to the judgment of ‘good quality’ wine. Latour et al. (2011) reported that less experienced wine consumers paid less attention to sensory experiences and were more influenced by advertisements when conducting a wine recognition task. In contrast, experts relied solely on their taste experience.

However, since appreciation and quality assessment involve not only sensory characteristics but also various cognitive, emotional, and affective dimensions (Peri, 2006), changes in perception driven by perceptual learning alone may not lead to enhanced appreciation and evaluation of quality. There are evidences that quality judgement and appreciation requires integration of both sensory experience and conceptual knowledge. Torri et al. (2013) suggested that wine experts, who possess more repeated exposure and extensive knowledge of wine, were better at distinguishing wines based on quality. This indicates that knowledge of wine, in addition to sensory perception, plays a crucial role in recognizing and evaluating quality. In the study by Lee et al. (2021), the group that received education, including lessons on the types and characteristics of black tea as well as tasting, was better able to distinguish high-quality black tea from lower-quality tea compared to the group that was merely exposed to black tea over the same period without any educational intervention. The educated group also actively utilized the floral and fruity notes, which are key characteristics of high-quality black tea, to construct an ideal representation. Latour et al. (2011) stated that novices are better able to assess wine quality when they first acquire conceptual knowledge and then activate it, as this allows them to leverage their perceptual experience in recognizing wines.

## Future studies

Perceptual learning in food context remains relatively unexplored in food context despite its significance. As mentioned previously, perceptual learning has been investigated predominantly in the field of wine research, likely stemming from the early recognition of perceptual learning that is responsible for differences in perception among wine experts, heavy users, and novices. Compared to the studies about wine, perceptual learning in other food categories remain insufficiently understood.

Several studies have suggested a link between perceptual learning and the development of liking through repeated experiences. However, the specific role of perceptual learning in these studies has not been explicitly elucidated. In particular, when intentional learning such as structured training or educational programs (e.g., wine or tea classes) is involved, it remains unclear whether the observed perceptual or hedonic changes result from perceptual learning, cognitive interventions, or both. Furthermore, if both mechanisms contribute, the nature of their interaction and how they collectively influence perception and liking require further investigation. Additionally, given that perceptual learning is characterized by long-term perceptual changes, it is essential to determine whether intentional learning has a lasting effect on sensory perception.

Another promising avenue for future studies is to explore whether the application of perceptual learning principles can be used to promote healthier dietary habits and encourage sustainable food choices. Repeated exposure has been shown to enhance familiarity and acceptance of novel foods, suggesting that perceptual learning could be leveraged to facilitate the adoption of more health-conscious and environmentally sustainable diets. For example, studies have suggested that repeated exposure to plant-based proteins and vegetables can improve consumer acceptance (de Wild et al., 2013; Appleton et al., 2018; Anzman-Frasca et al., 2012; Hoek et al., 2013; Akinmeye et al., 2024), potentially aiding the transition toward more sustainable dietary patterns. Moreover, structured interventions incorporating perceptual learning principles, such as guided tasting sessions designed to enhance top-down attention to specific sensory features, could help consumers develop a preference for nutrient-dense foods while reducing reliance on highly processed foods. Additionally, by identifying the sensory attributes to which individuals become more sensitive or adapt over time, targeted formulations or strategies could be developed to accelerate the formation of positive food preference. Given the increasing concerns over diet-related health issues and the environmental impact of food production, further empirical studies are needed to systematically explore how perceptual learning can be harnessed to influence dietary choices in a way that aligns with both public health objectives and sustainability goals.

One critical aspect that warrants further investigation is the ethical implications, particularly concerning the potential for consumer manipulation and disproportionate impacts on vulnerable populations. While insight into perceptual learning can be used to promote consumer engagement with healthier and more sustainable foods, interventions leveraging this principle may raise ethical concerns if deployed to promote the appeal of nutritionally poor foods. The potential to prime one’s food choice by unconsciously directing attention may limit consumer autonomy in food choices. Certain populations, such as children, individuals with lower nutrition literacy, and socioeconomically disadvantaged groups, may be particularly vulnerable to these influences. Another potential ethical concern is that studies on perceptual learning related to healthy or sustainable food choices are likely to be mostly conducted in Western, Educated, Industrialized, Rich, and Democratic (WEIRD) societies (Gómez-Corona, 2020; Gómez-Corona and Otterbring, 2025), raising concerns regarding the generalizability of these findings to underrepresented populations. The application of these findings to real-world interventions should consider cultural, economic, and social context to ensure equitable impact. Therefore, future studies should critically examine the ethical boundaries of its use.

In conclusion, perceptual learning, which develops through repeated experiences, including differentiation, unitization, attentional weighing, and stimulus imprinting, affects liking by increasing attention to salient features, while suppressing the irrelevant information. In the realm of food, perceptual learning leads to the heightened detection and identification of diverse sensory attributes and decreased background “noise” attributes in foods. Consequently, this modifies the sensory representation of food, influencing judgment with regard to liking, or altering a mental prototype or ideal which in turn shifts liking, appreciation, or quality assessments towards foods with more complex and nuanced flavors. In addition, perceptual learning can also contribute to the development of specific aversion or preference for certain flavors. However, unlike simpler solution systems based on taste or aroma compounds, it is challenging to isolate the role of perceptual learning in development of liking for foods from other factors, particularly cognitive inputs. Future research should delve deeper into the interplay between cognitive processes and perceptual learning, such as the interaction between explicit training and bottom-up sensory input in enhancing the appreciation and quality perception as well as the development of liking. Understanding these mechanisms could help in designing strategies not only to develop new high-end food products with sophisticated sensory profile but also to introduce novel foods, especially in promoting healthier, more sustainable food options, which currently encounter consumer resistance due to unfamiliarity.
